# DECT-based stratification of nonocclusive mesenteric ischemia using bowel-wall iodine concentration: a prospective single-center cohort

**DOI:** 10.1186/s41747-026-00760-9

**Published:** 2026-06-25

**Authors:** Bastien Roussel, Marine Julien, Romain Moinet, Anass El M’aary, Gabriel Simon, Celia Turco, Alexandre Doussot, Hadrien Winiszewski, Guillaume Besch, Gael Piton, Maxime Ronot, Paul Calame

**Affiliations:** 1 Department of Radiology, Université Marie et Louis Pasteur, CHU Besançon, Besançon, France; 2 Department of Digestive Surgery, Université Marie et Louis Pasteur, CHU Besançon, Besançon, France; 3 Medical Intensive Care Unit, Université Marie et Louis Pasteur, CHU Besançon, Besançon, France; 4 Surgical Intensive Care Unit, Université Marie et Louis Pasteur, CHU Besançon, Besançon, France; 5 Department of Radiology, Hôpital Beaujon, AP-HP.Nord, Université Paris Cité, INSERM Centre de Recherche sur l’Inflammation, Paris, France

**Keywords:** Iodine, Mesenteric ischemia (non-occlusive), Necrosis, Tomography (x-ray computed)

## Abstract

**Objective:**

We evaluated whether bowel-wall iodine concentration (BWIC) on dual-energy computed tomography (DECT) improves the diagnosis of non-occlusive mesenteric ischemia (NOMI) and allows differentiation of irreversible transmural necrosis (ITN) from non-ITN.

**Materials and methods:**

In this prospective single-center ethically approved study, consecutive patients with shock who underwent DECT between November 2022 and October 2024 were included. NOMI was classified as absent, present without ITN, or present with ITN using a composite reference standard. On 70- and 50-keV virtual monoenergetic imaging (VMI), one reader assessed signs of bowel ischemia. On iodine maps, BWIC was measured by placing five full-thickness bowel-wall regions of interest within the affected digestive segment(s). BWIC was measured by one reader and independently repeated by four additional readers in patients with NOMI. Diagnostic performance was assessed using the area under the curve (AUC) at receiver operating characteristic analysis and Youden J index.

**Results:**

Among 177 patients (median age 65 years; 44% males), NOMI was diagnosed in 31/177 (18%), including 23/31 (74%) with ITN. Absent enhancement on 50-keV VMI yielded the highest accuracy for NOMI diagnosis (J = 0.94) and was not outperformed by minimum BWIC (J = 0.87). In NOMI patients, no binary signs differentiated ITN from non-ITN. Minimum BWIC was lower in ITN (median 0.54 mgI/mL [interquartile range 0.42–0.60]) than in non-ITN (0.85 mgI/mL [0.74–0.99]; *p* < 0.001) with a pooled AUC of 0.86 and optimal threshold of 0.52 mgI/mL (J = 0.74).

**Conclusion:**

BWIC did not outperform visual assessment for diagnosing NOMI, but uniquely discriminated ITN from non-ITN.

**Relevance statement:**

Using a two-step strategy—50-keV-VMI bowel enhancement assessment followed by segmental BWIC measurement—correctly classified 173/177 patients as no NOMI, NOMI without or with irreversible transmural necrosis.

**Key Points:**

- Non-occlusive mesenteric ischemia (NOMI) affected 18% of shocked intensive care unit patients who underwent abdominal DECT.
- Absent bowel-wall enhancement at 50-keV virtual monoenergetic imaging best detected NOMI.
- Only bowel-wall iodine concentration discriminated transmural necrosis from reversible ischemia (pooled AUC = 0.86), with an optimal cutoff of 0.52 mgI/mL.

**Graphical Abstract:**

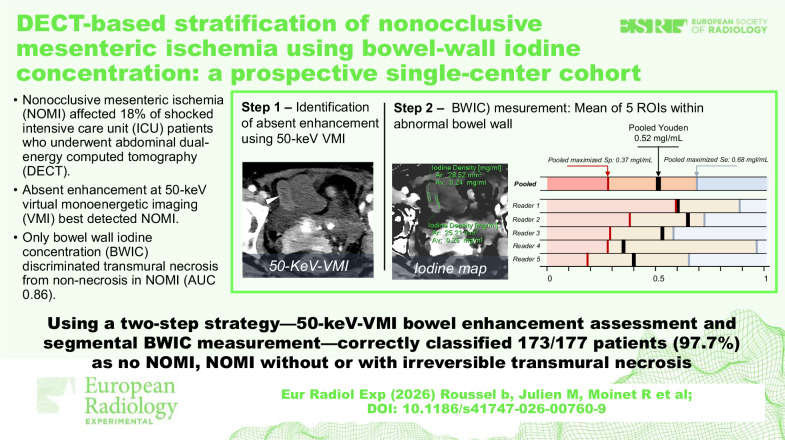

## Background

Non-occlusive mesenteric ischemia (NOMI) is a life-threatening complication of critical illness. It arises from profound splanchnic hypoperfusion in the absence of an obstructive arterial or venous lesion. Despite advances in resuscitation, organ support, and imaging, mortality remains 50–70% once irreversible transmural necrosis (ITN) develops [1, 2].

Early diagnosis is challenging because abdominal pain, ileus, and laboratory abnormalities are non‑specific [3] or masked by sedation and mechanical ventilation. Although biological assays such as the intestinal fatty acid–binding protein measurement are being investigated to advance noninvasive diagnosis [4, 5], abdominal computed tomography (CT) remains the first-line imaging modality, with reported sensitivities ranging from 60% to 80% [3, 6]. Several studies evaluating the ability of CT to diagnose ITN in NOMI have focused on the enhancement of the bowel wall, which is the most accurate CT feature in critical care settings, given the lower specificity of other CT features, such as bowel dilation or parietal pneumatosis [7–9].

However, relying on bowel-wall hypoenhancement to diagnose NOMI remains a risky strategy, because this subjective sign is prone to substantial interobserver variability, particularly when differentiating decreased from absent enhancement [3, 6, 9]. This limitation is critical because intestinal ischemia is not binary but evolves along a continuum from potentially reversible mucosal injury to transmural necrosis. In critically ill patients, interpretation is further complicated by motion artifacts, low cardiac output, and the frequent overlap between ischemic and nonischemic bowel abnormalities [3, 7]. In addition, CT performance is time-dependent, as early examinations may underestimate injury before overt enhancement defects appear.

Dual-energy CT (DECT) is an advanced technique that can provide additional information by estimating the iodine concentration within tissues. Applied to the bowel, this yields a quantitative parameter, namely the bowel-wall iodine concentration (BWIC) after intravenous administration of iodine-based contrast agent, which may enable an objective stratification of ischemia [10]. While promising results have been reported in small-bowel obstruction [11], studies in acute mesenteric ischemia are so far confined to improving diagnostic confidence [12–15] rather than diagnostic accuracy [16]. Importantly, no standardized method for BWIC measurement has yet been validated.

Thus, this study aimed at determining whether DECT, specifically through BWIC measurement, was superior to virtual monoenergetic reconstructions (VMI) for diagnosing NOMI and differentiating ITN from non-ITN cases in critically ill patients.

## Methods

### Patient selection and primary outcome

**Fig. 1:**
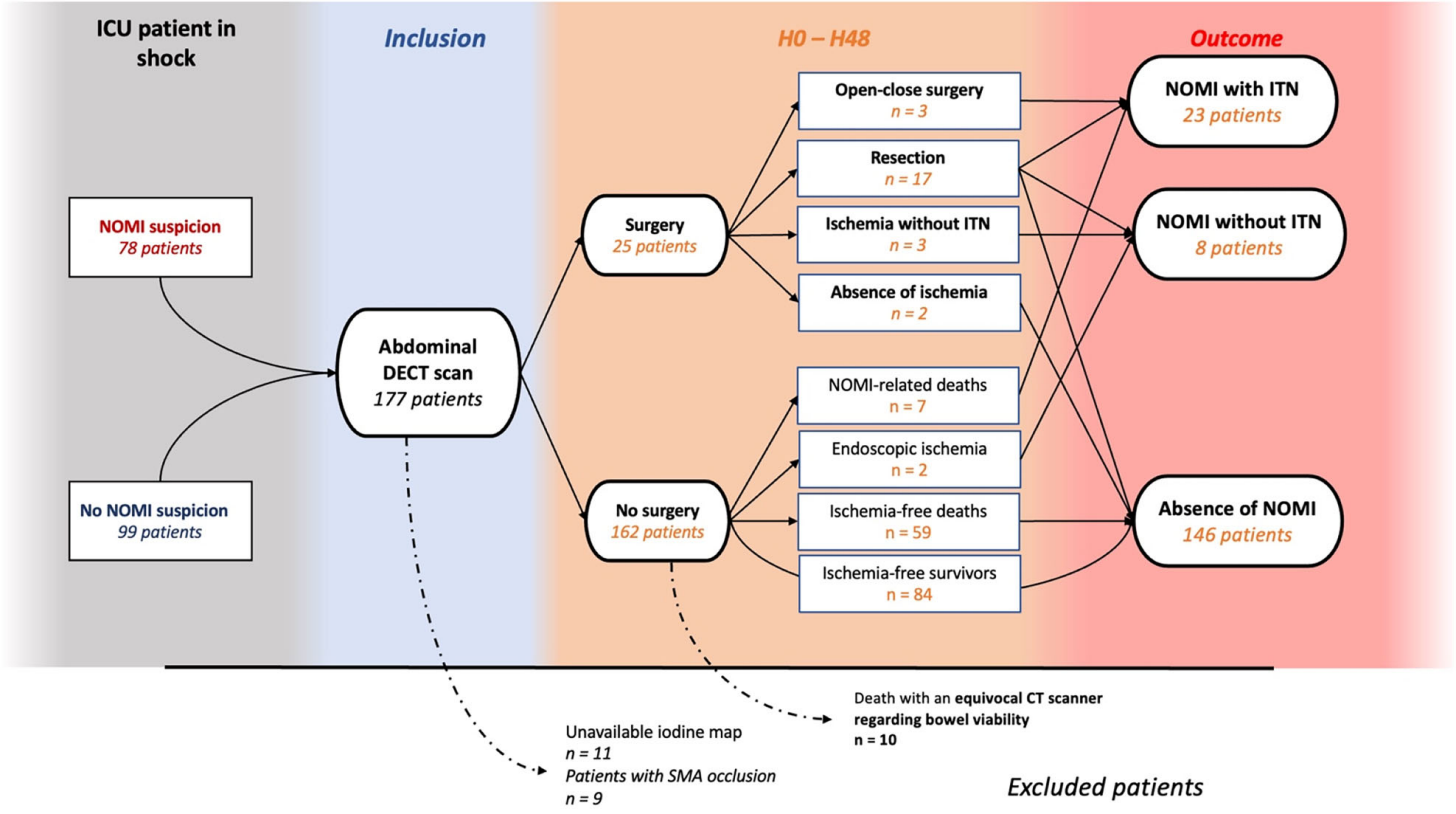
Flowchart of the study population. ICU, Intensive care unit; NOMI, Non-occlusive mesenteric ischemia; DECT, Dual-energy computed tomography; ITN, Irreversible transmural necrosis; SMA, Superior mesenteric artery

This study builds on a prospective observational cohort approved by our local institutional review board and supported by our institution. As this was an observational study with no modification of patient management, the requirement for informed consent was waived by the IRB (DRCI 2022/671). Consecutive patients in circulatory shock who were admitted to the intensive care unit of our institution and underwent abdominal DECT between November 2022 and October 2024 were prospectively included in our electronic database at the time of the DECT (Fig. 1).

Clinical and biological data were collected at the time of DECT. Follow-up data at 48 h were recorded for the assessment of NOMI. NOMI status (absent, present without ITN, or present with ITN) was determined using a composite reference standard that combined surgical, pathologic, endoscopic, and clinical outcome data within 48 h after DECT. ITN was defined as transmural ischemic necrosis involving at least the muscularis propria on pathology or, in nonresected cases, by unequivocal surgical or endoscopic evidence of nonviable bowel.

In patients who underwent laparotomy within 48 h following CT, surgical findings were recorded. In case of open-close surgery (*i.e*., absence of bowel resection given the extent of necrosis to the entire bowel), patients were diagnosed as NOMI with ITN. If resection were performed, pathology reports were collected, and the presence of ITN was reported. If resection was not performed but the surgeon observed lesions suggestive of bowel ischemia (diffuse bowel pallor associated with microhemorrhage), patients were considered as NOMI without ITN. If the surgeon observed any sign of bowel injury, a diagnosis of NOMI was considered for the composite reference standard.

In patients who did not undergo laparotomy, four scenarios were evaluated:

1. Death attributed to NOMI within the 48 h by the intensivist with evidence of ITN at CT examination and blood tests based on the previous NOMI-ITN score [3], patients were classified as NOMI with ITN;
2. Death within 48 h without any sign of bowel ischemic injury (*i.e*., absence of bowel dilation, bowel thickening, and normal bowel-wall enhancement), NOMI diagnosis was considered for the composite reference standard;
3. Patients with bowel endoscopy confirming ischemic mural lesions were classified as NOMI without ITN;
4. Death within 48 h associated with equivocal CT features regarding bowel viability, such as bowel dilatation, doubtful bowel-wall enhancement, bowel thickening, and the diagnosis of NOMI remained uncertain, as in its presence and as in its extension, patients were excluded.

Between November 2022 and October 2024, 207 ICU patients admitted for shock underwent an abdominal DECT at the study hospital. Patients with superior mesenteric artery occlusion (*n* = 9), without available iodine maps (*n* = 11), and patients who died within 48 h with an equivocal CT regarding bowel viability (*n* = 10) were excluded (Fig. 1).

### DECT protocol

All examinations were performed on a dual-layer DECT system (IQon Spectral CT, Philips Healthcare). Unenhanced images were first acquired, followed by contrast-enhanced acquisitions including an arterial phase triggered by bolus tracking in the aorta (threshold, 120 HU), and a portal venous phase acquired 80 s after the start of intravenous contrast injection. All patients received a total iodine dose of 600 mgI/kg body weight using iomeprol (Iomeron®, Bracco Imaging) at either 350 or 400 mgI/mL, administered with a power injector over a fixed duration of 30 s, followed by a saline flush. At our institution, iomeprol 350 mgI/mL is routinely used for standard abdominal CT (99/177, 56%), whereas iomeprol 400 mgI/mL is preferentially used when acute mesenteric ischemia is clinically suspected (78/177, 44%). Therefore, the total contrast volume and injection rate were not fixed values but depended directly on patient body weight and on the iodine concentration of the contrast agent (contrast volume of 1.71 mL/kg for iomeprol 350 mgI/mL and 1.50 mL/kg for iomeprol 400 mgI/mL, with corresponding injection rates of 0.057 mL/kg/s and 0.05 mL/kg/s, respectively). The resulting iodine delivery rate was identical in both groups, corresponding to 20 mgI/kg/s. Portal-venous phase images were reconstructed as 70-keV and 50-keV VMIs. The 70-keV VMI was considered the analogue of standard single-energy CT in routine practice, whereas 50-keV VMI was selected as the primary energy level for evaluating bowel-wall enhancement because, on this platform, 50 keV provides improved contrast-to-noise ratio for abdominal imaging while limiting the increase in image noise compared with lower-keV images. No oral contrast agent was administered.

### Qualitative image analysis

The first review of 70-keV VMI was prospective and performed by a single reader (Reader 1, B.R., with 4 years of experience in the field of abdominal imaging) on a dedicated workstation (Carestream Health), not blinded to all the clinical and biological tests. Following previous publications [3, 8], four CT features were prospectively collected: bowel-wall absent enhancement (AE), defined as the absence of bowel-wall enhancement compared to precontrast images or to normal adjacent bowel loops; thinned bowel wall, defined as virtual wall (*i.e*., nonvisible or paper-thin appearance); parietal pneumatosis; and bowel dilation, defined as a maximal bowel diameter > 25 mm.

### Quantitative image analysis

**Fig. 2:**
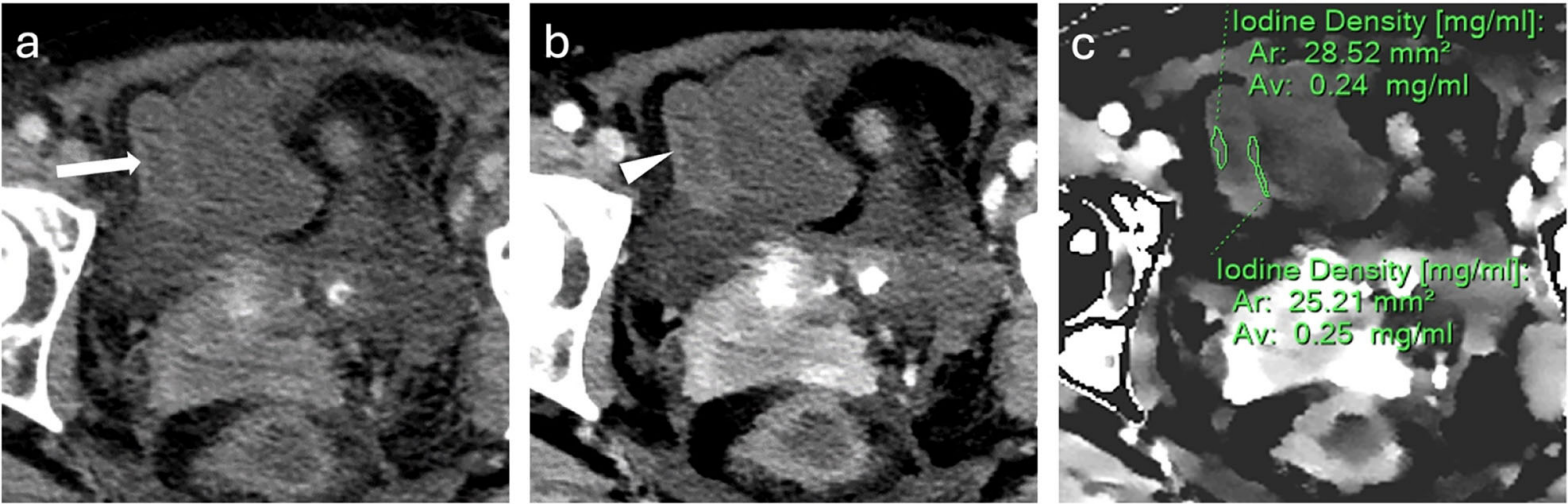
A 73-year-old woman admitted to the intensive care unit for necrotizing fasciitis of the lower limb, presenting with septic shock and abdominal pain following surgical debridement. Abdominal dual-energy computed tomography (DECT) was performed for suspected non-occlusive mesenteric ischemia (NOMI). **a** Axial 70-keV virtual monoenergetic imaging (VMI) shows an ileal loop in the pelvis with doubtful bowel-wall enhancement (arrow), but present. **b** Axial 50-keV VMI also demonstrates bowel-wall enhancement (arrowhead). **c** The iodine density map generated with DECT reveals very low iodine concentrations (0.24 and 0.25 mg/mL). Surgical exploration within 48 h resulted in the resection of the affected loop, with histopathological confirmation of irreversible transmural necrosis

In a second step, Reader 1 retrospectively and blindly reviewed DECT reconstructions. First, 50-keV VMI was assessed for bowel-wall enhancement. Then, iodine density maps from the portal venous phase were used for BWIC measurements (Fig. 2). ROI placement was performed as follows: (1) ROIs were manually drawn (area > 20 mm²) on the portal venous phase iodine density map; (2) five ROIs were distributed on consecutive axial slices within each evaluated bowel segment; (3) each ROI encompassed the full mural thickness while carefully excluding intraluminal contents, mural gas, adjacent mesenteric fat, and beam-hardening artifacts; and (4) partial-volume effects were minimized by excluding positions where the bowel wall was not reliably delineated. Segments in which the bowel wall was not reliably visible or was obscured by dense stool or severe artifacts were not measured.

**Fig. 3:**
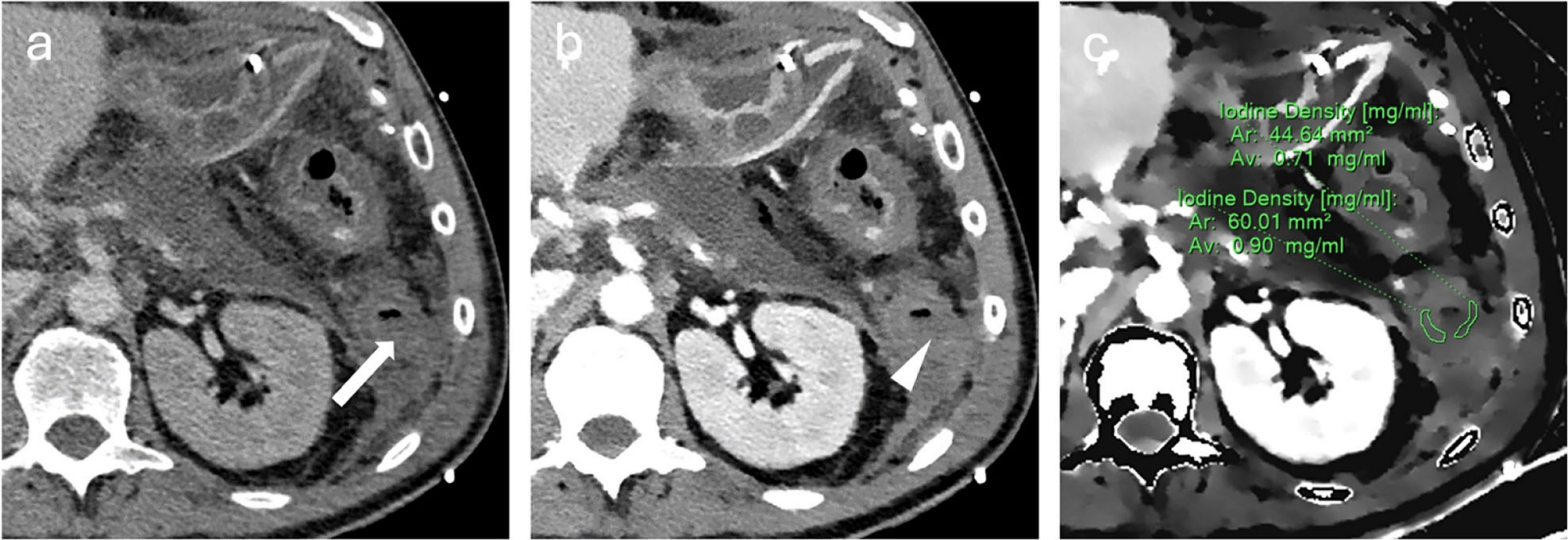
A 65-year-old man admitted to the intensive care unit for severe biliary acute pancreatitis (computed tomography (CT) severity index = 10), complicated by mesenteric ischemia requiring two ileocolic resections. The patient presented with hemodynamic and septic shock associated with abdominal pain. Abdominal dual-energy CT (DECT) was performed for suspected non-occlusive mesenteric ischemia (NOMI). **a** Axial 70-keV virtual monoenergetic imaging (VMI) shows the absence of enhancement of the bowel wall at the splenic flexure (arrow). **b** Axial 50-keV VMI confirms the lack of wall enhancement (arrowhead). **c** The iodine density map generated with DECT demonstrates residual iodine uptake within the bowel wall at the ROI locations (0.71 and 0.90 mgI/mL). Surgical exploration within 48 h led to resection of the affected bowel, and pathological examination showed an isolated mucosal necrosis

For Reader 1, BWIC was measured systematically in all evaluable bowel segments (jejunum, ileum, right colon, and left colon), with five ROIs placed per segment. The segmental mean BWIC was defined as the mean of the five ROI values. For each patient, the minimum BWIC corresponded to the lowest segmental mean BWIC, and the maximum BWIC corresponded to the highest segmental mean BWIC among all measured bowel segments. If a decreased wall enhancement suggesting bowel ischemia was depicted on 50-keV VMI (Fig. 3), these five ROIs were placed in the abnormal bowel segments.

To account for global hemodynamic status and contrast distribution, iodine concentrations were also measured in solid abdominal organs. For the liver, one circular ROI (100–150 mm²) was placed in the right lobe, avoiding visible vessels, bile ducts, and focal lesions. For the spleen and pancreas, one ROI of similar size was placed in homogeneous parenchyma, avoiding vessels and focal lesions. All organ ROIs were placed on the same portal-venous phase iodine maps used for bowel measurements.

To evaluate the feasibility and reproducibility of the BWIC measurement, a subgroup analysis was retrospectively performed in patients with a confirmed NOMI. Four readers (three junior abdominal radiologists, A.E.M., R.M., and M.J., with 4, 5, and 5 years’ experience, respectively, and one senior radiologist with 13 years’ experience, P.C.) were asked to assess 70-keV-VMI and 50-keV-VMI AE, and to manually draw five ROIs (> 20 mm²) in visually abnormal bowel wall segments using the same ROI placement methodology. Image interpretation was performed in two separate sessions: a conventional CT session based on 70-keV VMI alone, and a DECT session based on 50-keV VMI reviewed jointly with portal-venous-phase iodine maps. Readers were fully blinded to clinical outcomes, NOMI status, and Reader 1’s measurements.

### Statistical analysis

Continuous variables were expressed as median [IQR] and mean ± standard deviation (SD) and compared with the Mann–Whitney *U* test or Student’s test as appropriate; categorical variables were reported as numbers and percentages and compared using Fisher exact test or χ^2^ as appropriate. The diagnostic performance of the BWIC was assessed in two steps.

First, for the diagnosis of NOMI (based on Reader 1), receiver operating characteristic analysis was performed. The optimal threshold was determined by maximizing Youden index (J = sensitivity *plus* specificity *minus* 1). At this cutoff, sensitivity, specificity, positive predictive value, negative predictive value, and overall accuracy were calculated, each with 95% confidence interval (CI).

Second, for the diagnosis of ITN in patients with NOMI, receiver operating characteristic analysis was performed using data from Readers 1 to 5. The area under the curve (AUC) and 95% CIs were estimated using DeLong’s method. The cutoff that maximized the Youden index was selected and applied to compute sensitivity, specificity, negative and positive predictive value, and overall accuracy, each with 95% CI. The same approach was applied to the three dichotomous CT signs recorded by each reader. No formal sample size calculation was performed because no prior data were available to support a reliable estimate of effect size at study initiation.

Finally, inter-reader agreement for BWIC measurements was evaluated using the intraclass correlation coefficient (ICC). Missing-data rates were < 5% for all clinical and biochemical variables. All tests were two-tailed; *p* < 0.05 was considered statistically significant. Analyses were performed with R 4.3.2 (R Foundation, Vienna, Austria). The study was reported in accordance with the STARD 2015 guidelines.

## Results

### Study population

Among the 177 included patients, 77 were men (44%), and the median age was 65 years (interquartile range, IQR: 56–73). Among the 31 patients classified as having NOMI, diagnosis was supported by laparotomy in 3/31 (10%), histopathology of resected bowel specimens in 19/31 (61%), endoscopy in 2/31 (6%), and by concordant clinical and imaging criteria within 48 h in 7/31 (23%). ITN was confirmed by pathology in 13/23 (57%) patients, by open-close surgery in 3/23 (13%), and in 7/23 (30%) by death attributed to NOMI within 48 h with concordant imaging findings. Bowel ischemia was localized to the jejunum in 8/31 patients (26%), the ileum in 14/31 (45%), the right colon in 12/31 (39%), and the left colon in 18/31 (58%). Bowel involvement was confined to a single segment in 20/31 cases (65%), whereas 11/31 patients (35%) had multifocal disease involving two or more segments.

Regarding the etiology of circulatory shock, 79/177 (45%) had sepsis, 35/177 (20%) hemorrhagic shock, 25/177 (14%) cardiogenic shock, and 26/177 (15%) a postoperative status. Continuous infusion of norepinephrine was required in 130/177 (73%) patients (median dose during CT scanning 0.6 µg·kg⁻¹·min⁻¹ [IQR 0.0–2.5]), and 103/177 (58%) were invasively mechanically ventilated at the time of CT scanning.

Among the 177 DECT examinations performed in the ICU, clinical suspicion of NOMI was the leading indication (78/177, 44%) followed by septic shock (46/177, 26%), active bleeding (28/177, 16%) investigations, unexplained hemodynamic instability (11/177, 6%), routine postoperative control (8/177, 5%), assessment after out-of-hospital or in-hospital cardiac arrest (5/177, 3%), and acute intoxication (2/177, 1%).

Among the 21 patients with NOMI who underwent surgery, the median time interval was 6 [IQR 4–24] hours. Median delay between CT and surgery in patients with NOMI without ITN was 10 [IQR 4–28] hours compared to 6 [IQR 4–24] hours in patients with ITN (*p* = 0.943). Among the 31 patients with NOMI, 23/31 (74%) died within 28 days (9 at day 1, 3 at day 2, and 11 within 28 days).

### Variables associated with NOMI

Baseline clinical and biological characteristics stratified by NOMI diagnosis are detailed in Table 1. Table 2 shows the imaging findings associated with NOMI. 70-keV-VMI and 50-keV-VMI AE were the most discriminant imaging features (both *p* < 0.001). Small-bowel dilatation was observed in 16/31 (52%) patients with NOMI *versus* 23/146 (16%) controls (*p* < 0.001). Parietal pneumatosis was present in 6/31 (19%) NOMI cases compared with 1/146 (1%) controls (*p* < 0.001), while portal venous gas was detected in 5/31 (16%) *versus* 0/146 (0%) patients, respectively (*p* < 0.001).

**Table 1:** Clinical and biological variables associated with the diagnosis of non-occlusive mesenteric ischemia

	NOMI present	NOMI absent	
	(*n* = 31)	(*n* = 146)	*p*-value
Patient characteristics			
Age, years	68.0 [62.0–73.0]	65.0 [56.0–72.8]	0.202
Male sex	9/31 (29%)	68/146 (47%)	0.110
Hypertension	22/30 (73%)	68/138 (49%)	0.025
Dyslipidemia	15/30 (50%)	48/138 (35%)	0.146
Body mass index, kg/m²	26.6 [22.9–30.6]	26.6 [23.0–31.6]	0.893
Diabetes mellitus	6/30 (20%)	42/137 (31%)	0.275
Cardiovascular comorbidities	17/30 (57%)	67/138 (49%)	0.546
Chronic kidney disease	4/30 (13%)	22/137 (16%)	1.000
Etiology of shock			
Sepsis	13/30 (43%)	66/137 (48%)	0.689
Hemorrhagic shock	6/30 (20%)	29/137 (21%)	1.000
Cardiogenic shock	3/30 (10%)	22/137 (16%)	0.574
Postoperative status	8/30 (27%)	18/137 (13%)	0.092
Clinical variables			
Heart rate, beats per minute	101.5 [90.5–116.8]	97.0 [80.0–115.0]	0.195
Respiratory rate, /min	25.0 [18.0–31.0]	21.0 [18.0–25.0]	0.080
Systolic arterial pressure, mmHg	105.0 [98.0–122.0]	110.0 [95.2–130.8]	0.624
Diastolic arterial pressure, mmHg	50.0 [42.0–57.0]	60.0 [50.0–65.8]	0.007
Norepinephrine dose, µg/kg/min	2.1 [0.1–4.0]	0.7 [0.0–2.5]	0.069
Invasive mechanical ventilation	1.0 [0.2–1.0]	1.0 [0.0–1.0]	0.215
Body temperature, °C	36.8 [36.2–37.8]	36.8 [36.2–37.5]	0.836
Glasgow Coma Scale	15.0 [14.0–15.0]	15.0 [8.0–15.0]	0.144
Enteral nutrition	8/28 (29%)	28/140 (20%)	0.320
Parenteral nutrition	5/27 (19%)	14/140 (10%)	0.198
Blood tests			
Serum lactate, mmol/L	4.9 [2.4–7.2]	2.6 [1.6–4.2]	0.002
Bicarbonate, mmol/L	16.0 [13.5–22.0]	19.0 [16.0–23.0]	0.054
PaO_2_, kPa	11.8 [9.1–14.8]	11.4 [9.5–14.7]	0.972
PaCO_2_, kPa	4.5 [4.0–5.3]	4.5 [3.8–5.4]	0.998
Arterial pH	7.3 [7.2–7.4]	7.4 [7.3–7.4]	0.011
AST, U/L	140.0 [61.0–828.0]	84.0 [39.0–240.5]	0.012
ALT, U/L	151.0 [45.0–421.0]	54.0 [25.0–147.0]	0.010
Total bilirubin, µmol/L	18.3 [11.8–43.1]	13.1 [8.0–30.0]	0.050
Hemoglobin, g/dL	9.0 [8.3–11.4]	9.6 [8.2–11.7]	0.835
Platelet count, × 10⁹/L	160.0 [92.5–252.5]	183.0 [102.0–248.0]	0.676
Prothrombin time, %	48.0 [29.5–67.0]	64.0 [47.0–79.2]	0.001
Serum creatinine, µmol/L	145.0 [101.0–238.5]	115.0 [80.0–200.0]	0.076
SOFA score at CT	12.0 [9.5–13.5]	9.0 [7.0–11.0]	0.023
SAPS II score	61.0 [51.0–74.0]	54.0 [42.0–68.0]	0.023

Data are given as median [interquartile range] or ratio (percentage)

*NOMI* Non-occlusive mesenteric ischemia, *SOFA* Sequential organ failure assessment, *SAPS* Simplified acute physiology score, *AST* Aspartate aminotransferase, *ALT* Alanine aminotransferase

**Table 2:** Imaging variables associated with non-occlusive mesenteric ischemia

Variable	NOMI present	NOMI absent	*p*-value
	(*n* = 31)	(*n* = 146)	
AE 70-keV-VMI			
Any segment	29/31 (94%)	3/146 (2%)	< 0.001
Jejunum	8/31 (26%)	0/146 (0%)	< 0.001
Ileum	19/31 (61%)	0/146 (0%)	< 0.001
Left colon	6/28 (21%)	0/141 (0%)	< 0.001
Ascending colon	11/31 (35%)	3/145 (2%)	< 0.001
AE 50-keV-VMI			
Any segment	29/31 (94%)	0/146 (0%)	< 0.001
Bowel dilatation			
Any small-bowel dilatation	16/31 (52%)	23 146 (16%)	< 0.001
Jejunal dilatation	13/31 (42%)	19/146 (13%)	< 0.001
Maximal diameter, mm	25.0 [19.5–32.5]	18.5 [15.2–23.0]	< 0.001
Ileal dilatation	13/31 (42%)	13/146 (9%)	< 0.001
Maximal diameter, mm	22.0 [16.5–27.5]	13.0 [10.0–16.8]	< 0.001
Pneumatosis intestinalis			
Any segment	6/31 (19%)	1/146 (1%)	< 0.001
Jejunal pneumatosis	6/31 (19%)	0/146 (0%)	< 0.001
Ileal pneumatosis	3/31 (10%)	0/146 (0%)	0.005
Descending colon pneumatosis	1/28 (4%)	1/141 (1%)	0.305
Ascending colon pneumatosis	0/31 (0%)	0/145 (0%)	1.000
Portal venous gas	5/31 (16%)	0/146 (0%)	< 0.001
Splenic infarcts	17/31 (55%)	26/145 (18%)	< 0.001
Diffuse kidney infarction	14/31 (45%)	21/146 (14%)	< 0.001
Iodine concentration variable			
Mean BWIC_mean_ (mgI/mL)	1.23 [1.08–1.55]	1.92 [1.64–2.26]	< 0.001
Minimum BWIC_mean_ (mgI/mL)	0.60 [0.44–0.75]	1.44 [1.20–1.81]	< 0.001
Maximum BWIC_mean_ (mgI/mL)	1.98 [1.67–2.60]	2.50 [2.04–2.93]	0.003
BWIC_mean_ jejunum (mgI/mL)	1.82 [0.80–2.37]	2.42 [1.95–2.91]	< 0.001
BWIC_mean_ ileum (mgI/mL)	0.70 [0.53–1.45]	1.95 [1.58–2.37]	< 0.001
BWIC_mean_ right colon (mgI/mL)	1.42 [0.93–1.73]	1.71 [1.40–2.15]	0.002
BWIC_mean_ left colon (mgI/mL)	1.06 [0.76–1.35]	1.59 [1.29–1.96]	< 0.001
Pancreatic iodine concentration (mgI/mL)	2.76 [2.25–3.08]	2.91 [2.48–3.52]	0.090
Splenic iodine concentration (mgI/mL)	2.78 [2.18–3.37]	3.26 [2.77–3.94]	0.004
Liver iodine concentration (mgI/mL)	2.54 [2.06–3.27]	2.82 [2.27–3.41]	0.160

Data are given as median [interquartile range] or ratio (percentage)

*NOMI* Non-occlusive mesenteric ischemia, *BWIC* Bowel-wall iodine concentration, *AE* Absent enhancement, *VMI* Monoenergetic imaging

**Fig. 4:**
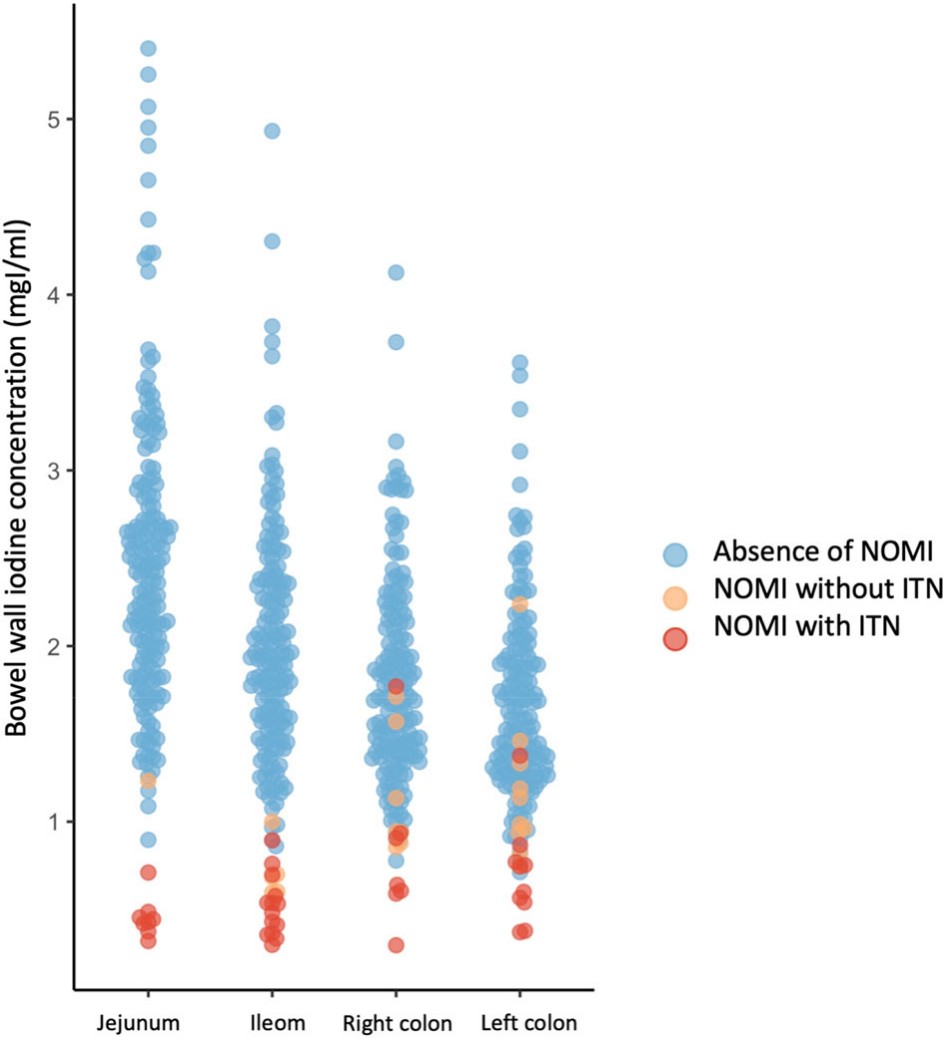
Scatter plot showing mean bowel-wall iodine concentration (BWIC, mg/mL) calculated from five regions of interest per bowel segment, stratified by the presence of non-occlusive mesenteric ischemia (NOMI) and by irreversible transmural necrosis status

Regarding quantitative analyses, the minimum mean BWIC (*i.e*., the lowest segmental mean BWIC measured for a given patient) was significantly lower in NOMI patients (0.60 mgI/mL [IQR 0.44–0.75] *versus* 1.44 mgI/mL [IQR 1.20–1.81]; *p* < 0.001). The BWICmean was significantly lower in NOMI patients across all measured regions of the gastrointestinal tract (Fig. 4). This was particularly notable in the jejunum (1.82 mgI/mL [IQR 0.80–2.37] *versus* 2.42 mgI/mL [1.95–2.91]; *p* < 0.001) and the ileum (0.70 mgI/mL [0.53–1.45] *versus* 1.95 mgI/mL [1.58–2.37]; *p* < 0.001), with similarly significant reductions observed in both right and left colon segments (all *p* < 0.001). In contrast, the iodine concentrations in the liver (*p* = 0.160) and the pancreas (*p* = 0.090) were not significantly different between groups.

A minimum mean BWIC cutoff of ≤ 1.0 mgI/mL yielded 97% sensitivity (95% CI 83–100%) and 90% specificity (95% CI 84–95%), with an accuracy of 92% and a Youden J index of 0.87 for the diagnosis of NOMI (Table 3).

**Table 3:** Diagnostic performances of imaging features to diagnose non-occlusive mesenteric ischemia

	Cutoff*	TP/FP/TN/FN	Sensitivity % (95% CI)	Specificity % (95% CI)	PPV % (95% CI)	NPV % (95% CI)	Accuracy % (95% CI)	Youden J
Minimum BWIC_mean_	≤ 1 mgI/mL	30/14/132/1	97 (84–99)	90 (85–94)	68 (53–80)	99 (96–100)	92 (87–95)	0.87
AE at 70 keV-VMI	—	29/3/143/2	94 (79–98)	98 (94–99)	91 (76–97)	99 (95–100)	97 (94–99)	0.91
AE at 50 keV-VMI	—	29/0/146/2	94 (79–98)	100 (97–100)	100 (88–100)	99 (95–100)	99 (96–100)	0.94
Thinned bowel wall	—	10/0/146/21	32 (19–50)	100 (97–100)	100 (72–100)	87 (82–92)	88 (83–92)	0.32
Small-bowel dilatation	—	16/23/123/15	52 (35–68)	84 (77–89)	41 (27–57)	89 (83–93)	79 (72–84)	0.36
Parietal pneumatosis	—	6/1/145/25	19 (9–36)	99 (96–100)	86 (49–97)	85 (79–90)	85 (79–90)	0.19
Portal venous gas	—	5/0/146/26	16 (7–33)	100 (97–100)	100 (57–100)	85 (79–89)	85 (79–90)	0.16
Splenic infarcts	—	17/26/119/14	55 (38–71)	82 (75–87)	40 (26–54)	89 (83–94)	77 (71–83)	0.37
Diffuse kidney infarction	—	14/21/125/17	45 (29–62)	86 (79–90)	40 (26–56)	88 (82–92)	79 (72–84)	0.31

*BWIC*_*mean*_ Mean bowel-wall iodine concentration, *AE* Absent enhancement, *AUC* Area under the curve, *CI* Confidence interval, *FN* False negative, *FP* False positive, *NPV* Negative predictive value, *PPV* Positive predictive value, *TN* True negative, *TP* True positive, *VMI* Monoenergetic imaging

### Diagnosis of ITN in patients with NOMI

Clinical, laboratory, and imaging variables associated with ITN in patients with NOMI are summarized in Table 4. Patients with ITN tended to receive higher doses of norepinephrine (3.0 µg/kg/min [0.9–4.5] *versus* 0.3 [0–2.0], *p* = 0.092). Among laboratory variables, only serum bicarbonate levels differed significantly, being lower in the ITN group (15 mmol/L [12–20] *versus* 22 mmol/L [18–26], *p* = 0.016). Morphologic CT findings, such as the AE (both on 50-keV-VMI and 70 keV-VMI), small-bowel dilatation, and portal venous gas, were not associated with the presence of ITN. The minimum mean BWIC was the only quantitative imaging parameter significantly associated with ITN: 0.54 mgI/mL [IQR 0.42–0.60] *versus* 0.85 [0.74–0.99], *p* < 0.001.

**Table 4:** Variables associated with irreversible transmural necrosis in patients with non-occlusive mesenteric ischemia

	Univariate analysis			Multivariate analysis		
Variable	NOMI with ITN (*n* = 23)	NOMI without ITN (*n* = 8)	*p*-value	OR	95% CI	*p*-value
Clinical variables						
Norepinephrine dose, µg/kg/min	3.0 [0.9–4.5]	0.3 [0–2.0]	0.092	1.04	0.71–1.50	0.855
Blood test variables						
Serum lactate, mmol/L	5.1 [2.6–8.3]	4.3 [1.5–5.8]	0.168	—	—	—
Bicarbonate, mmol/L	15 [12–20]	22 [18–26]	0.016	0.98	0.78–1.24	0.879
AST, U/L	159 [75–828]	87 [51–1115]	0.453	—	—	—
ALT, U/L	243 [58–421]	52 [25–548]	0.302	—	—	—
Prothrombin time, %	50 [30–64]	39 [17–70]	0.701	—	—	—
Computed tomography-related variables						
AE 70-keV-VMI	22/23 (96)	7/8 (88)	0.456	—	—	—
AE 50-keV-VMI	23/23 (100)	6/8 (75)	0.060	—	—	—
Small-bowel dilatation	14/23 (61)	2/8 (25)	0.113	—	—	—
Jejunal dilatation	12/23 (52)	1/8 (12)	0.095	—	—	—
Maximal jejunal diameter, mm	27 [22–36]	21 [19–26]	0.067	—	—	—
Ileal dilatation	11/23 (48)	2/8 (25)	0.412	—	—	—
Maximal ileal diameter, mm	26 [16–28]	20 [17–24]	0.498	—	—	—
Pneumatosis intestinalis	6/23 (26)	0/8 (0)	0.298	—	—	—
Portal venous gas	5/23 (22)	0/8 (0)	0.291	—	—	—
Diffuse kidney infarction	10/23 (43)	4/8 (50)	1.000	—	—	—
Iodine concentration variables						
Mean BWIC, mgI/mL	1.536 [1.15–1.87]	1.234 [1.06–1.49]	0.101	—	—	—
Minimum BWIC_mean_, mgI/mL	0.54 [0.42–0.60]	0.85 [0.74–0.99]	< 0.001	2.73†	1.03–7.23	0.044
Maximum BWIC_mean_, mgI/mL	1.98 [1.67–2.60]	2.17 [1.76–2.53]	0.774	—	—	—
Hepatic iodine concentration, mgI/mL	2.52 [2.14–3.10]	2.61 [2.00–3.85]	0.696	—	—	—
Pancreatic iodine concentration, mgI/mL	2.76 [2.31–3.08]	2.44 [2.21–2.91]	0.701	—	—	—
Splenic iodine concentration, mgI/mL	2.78 [1.99–3.34]	2.86 [2.50–3.42]	0.494	—	—	—

Data are median [interquartile range] or number of patients, with percentages in parentheses. Multivariable logistic regression was performed with irreversible transmural necrosis as the dependent variable and included minimum BWIC_mean_, norepinephrine dose, and bicarbonate level

*AE* Absent enhancement, *BWIC* Bowel-wall iodine concentration, *ITN* Irreversible transmural necrosis, *NOMI* Non-occlusive mesenteric ischemia, *OR* Odds ratio, *VMI* Monoenergetic imaging

† OR expressed per 0.1 mgI/mL decrease in minimum BWIC_mean_

**Fig. 5:**
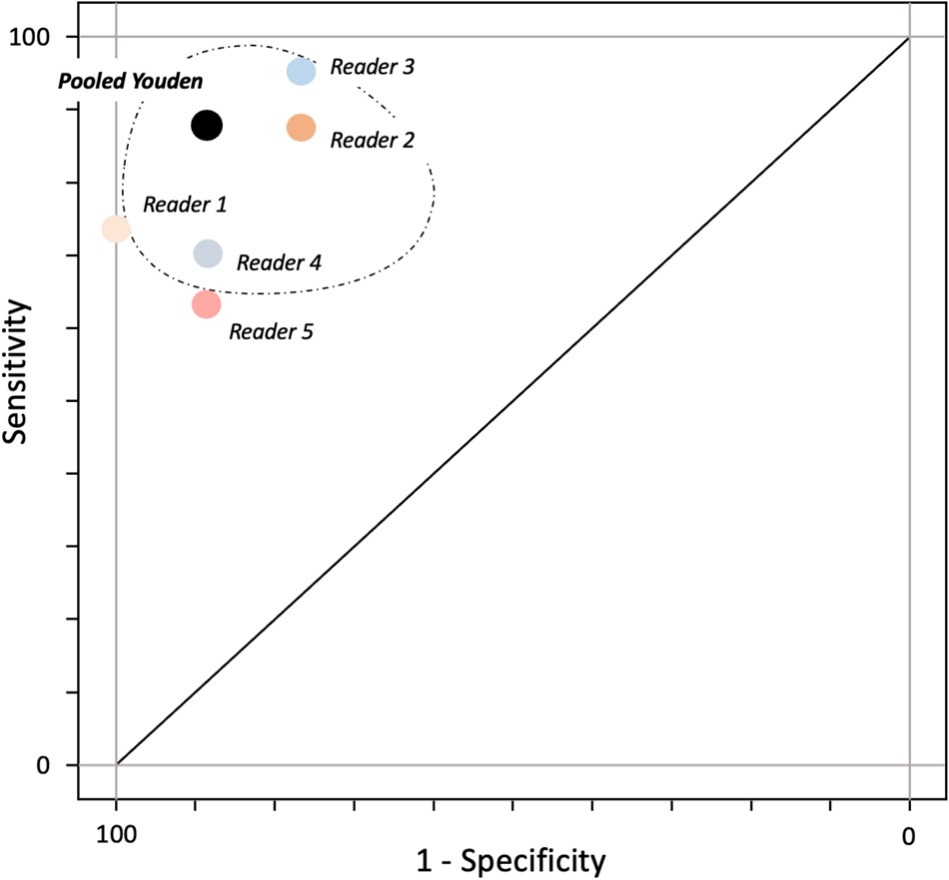
Horizontal bar plots showing the mean bowel-wall iodine concentration values measured by each reader in patients with non-occlusive mesenteric ischemia. For each reader, the vertical red line represents the threshold maximizing sensitivity, the blue line the threshold maximizing specificity, and the black line the threshold maximizing Youden J index. The pooled analysis (upper bar) yielded optimal thresholds of 0.36 mgI/mL for sensitivity, 0.65 mgI/mL for specificity, and 0.52 mgI/mL for Youden J index

The diagnostic performances of DECT parameters for identifying ITN are summarized in Table 5, based on interpretation by five independent readers. The minimum mean BWIC demonstrated strong diagnostic performance, with sensitivity ranging from 65% to 96% and specificity from 75% to 100%. Corresponding AUC values ranged from 0.74 to 0.93, with Youden J indices up to 0.74. The optimal minimum mean BWIC cutoff values varied between 0.36 and 0.65 mgI/mL for identifying ITN in patients with NOMI. In a pooled analysis, the optimal mean BWIC threshold for diagnosis of ITN was 0.52 mgI/mL (Youden J index = 0.74), with additional cutoffs of 0.36 mgI/mL, which maximized sensitivity, and 0.65 mgI/mL, which maximized specificity (Fig. 5). Sensitivity analysis excluding the 7 patients who died without ITN confirmation is presented in Supplementary Table S1.

**Table 5:** Diagnostic performance of DECT to diagnose irreversible transmural necrosis in non-occlusive mesenteric ischemia

		AUC	Optimal cutoff*	TP/FP/TN/FN	Sensitivity (95% CI)	Specificity (95% CI)	PPV (95% CI)	NPV (95% CI)	Accuracy (95% CI)	Youden
Reader 1 (4 years of experience)	BWIC_mean_	0.93 (0.84–1.00)	≤ 0.60	17/0/8/6	73 (52–87)	100 (68–100)	100 (82–100)	57 (33–79)	81 (65–92)	0.74
	Max Se		≤ 0.89	23/5/3/0	100 (86–100)	38 (14–69)	82 (64–92)	100 (44–100)	84 (67–93)	0.38
	Max Sp		≤ 0.60	17/0/8/6	74 (54–87)	100 (68–100)	100 (82–100)	57 (33–79)	81 (64–91)	0.74
	70 keV-VMI AE			21/8/0/2	91 (73–98)	0 (0–32)	72 (54–85)	0 (0–66)	68 (50–81)	-0.09
	50 keV-VMI AE			23/6/2/0	100 (86–100)	25 (7–59)	79 (62–90)	100 (34–100)	81 (64–91)	0.25
Reader 2 (5 years of experience)	BWIC_mean_	0.91 (0.81–1.00)	≤ 0.65	20/2/6/3	87 (68–95)	75 (41–93)	91 (72–97)	67 (35–88)	84 (67–93)	0.62
	Max Se		≤ 0.72	23/8/0/0	100 (86–100)	0 (0–32)	74 (57–86)	—	74 (57–86)	0.00
	Max Sp		≤ 0.49	12/0/8/11	52 (33–71)	100 (68–100)	100 (76–100)	42 (23–64)	65 (47–79)	0.52
	70 keV-VMI AE			20/7/1/3	87 (68–95)	13 (2–47)	74 (55–87)	25 (5–70)	68 (50–81)	-0.01
	50 keV-VMI AE			22/7/1/1	96 (79–100)	0 (0–32)	76 (58–88)	50 (9–91)	74 (57–86)	0.00
Reader 3 (13 years of experience)	BWIC_mean_	0.86 (0.73–0.99)	≤ 0.53	22/2/6/1	96 (79–100)	75 (41–93)	92 (74–98)	86 (49–97)	90 (75–97)	0.71
	Max Se		≤ 0.58	23/4/4/0	100 (86–100)	50 (22–78)	85 (68–94)	100 (51–100)	87 (71–95)	0.50
	Max Sp		≤ 0.39	13/0/8/10	57 (37–74)	100 (68–100)	100 (77–100)	44 (25–66)	68 (50–81)	0.57
	70 keV-VMI AE			20/5/3/3	87 (68–95)	38 (14–69)	80 (61–91)	50 (19–81)	74 (57–86)	0.25
	50 keV-VMI AE			23/8/0/0	100 (86–100)	0 (0–32)	74 (57–86)	—	74 (57–86)	0.00
Reader 4 (5 years of experience)	BWIC_mean_	0.78 (0.61–0.95)	≤ 0.36	16/1/7/7	70 (49–84)	88 (53–98)	94 (73–99)	50 (27–73)	74 (57–87)	0.57
	Max Se		≤ 0.97	23/7/1/0	100 (86–100)	12 (2–47)	77 (59–88)	100 (21–100)	77 (60–89)	0.12
	Max Sp		≤ 0.28	9/0/8/14	39 (22–59)	100 (68–100)	100 (70–100)	36 (20–57)	55 (38–71)	0.39
	70 keV-VMI AE			21/6/2/2	91 (73–98)	25 (7–59)	78 (59–89)	50 (15–85)	74 (57–86)	0.16
	50 keV-VMI AE			22/6/2/1	96 (79–100)	25 (7–59)	79 (60–90)	67 (21–94)	77 (60–88)	0.21
Reader 5 (3 years of experience)	BWIC_mean_	0.74 (0.56–0.92)	≤ 0.40	15/1/7/8	65 (45–81)	88 (53–98)	94 (72–99)	47 (25–70)	71 (53–84)	0.53
	Max Se		≤ 0.65	23/5/3/0	100 (86–100)	38 (14–69)	82 (64–92)	100 (44–100)	84 (67–93)	0.38
	Max Sp		≤ 0.19	2/0/8/21	9 (2–27)	100 (68–100)	100 (34–100)	28 (15–46)	32 (19–50)	0.09
	70 keV-VMI AE			14/5/3/9	61 (42–78)	38 (14–69)	74 (51–88)	25 (9–53)	55 (38–72)	-0.02
	50 keV-VMI AE			23/8/0/0	100 (86–100)	0 (0–32)	74 (57–86)	—	74 (57–86)	0.00
Pooled (5-reader mean)	Youden	0.86 (0.73–0.99)	≤ 0.52	20/1/7/3	87 (68–95)	88 (53–98)	95 (77–99)	70 (40–89)	87 (71–95)	0.74
	Max Se		≤ 0.68	23/5/3/0	100 (86–100)	38 (14–69)	82 (64–92)	100 (44–100)	84 (67–93)	0.38
	Max Sp		≤ 0.39	8/0/8/15	35 (19–55)	100 (68–100)	100 (68–100)	35 (19–55)	52 (35–68)	0.35

Data are percentages, with 95% confidence intervals in parentheses. For BWIC_mean_, the optimal cutoff = threshold defined by the Youden J index. Max Se = Threshold maximizing sensitivity. Max Sp = Threshold maximizing specificity. Pooled analysis = Mean BWIC across the 5 readers

*AE* Absent enhancement, *AUC* Area under the curve, *BWIC* Bowel-wall iodine concentration, *CI* Confidence interval, *FN* False negative, *FP* False positive, *ITN* Irreversible transmural necrosis, *Max Se* Maximum sensitivity, *Max Sp* Maximum specificity, *NOMI* Non-occlusive mesenteric ischemia, *NPV* Negative predictive value, *PPV* Positive predictive value, *TN* True negative, *TP* True positive, *VMI* Virtual monoenergetic imaging

### Inter-reader reproducibility of BWIC measurement

Inter-observer reliability for BWIC was moderate with an overall absolute-agreement ICC(A,1) of 0.52 (31 subjects, 5 readers; 95% CI 0.32–0.70). Consistency was higher, with an overall ICC(C,1) of 0.62 (95% CI: 0.47–0.72, bootstrap), suggesting the presence of systematic inter-reader differences. Reader-specific mean pairwise absolute-agreement ICCs were 0.62 (95% CI: 0.49–0.71) for Reader 1, 0.61 (95% CI: 0.42–0.72) for Reader 2, 0.49 (95% CI: 0.26–0.64) for Reader 3, 0.49 (95% CI: 0.31–0.60) for Reader 4 and 0.49 (95% CI: 0.37–0.58) for Reader 5. The strongest inter-reader agreement was observed for Reader 1–Reader 4 with ICC(A,1) = 0.75 (95% CI: 0.58–0.84), whereas the weakest was Reader 4–Reader 5 with ICC(A,1) = 0.36 (95% CI: 0.25–0.45); the arithmetic mean across all ten dyads was 0.54 (95% CI: 0.39–0.64). Inter-reader associations were strongest using pairwise Pearson correlations, ranging from 0.50 to 0.82 (mean 0.66, 95% CI: 0.49–0.78).

### Comparative performance of VMI and BWIC for diagnosing NOMI and ITN

In the whole cohort, Youden’s J for diagnosing NOMI was 0.94 (95% CI: 0.83–1.00) for the 50 keV-VMI AE, 0.91 (95% CI: 0.81–0.99) for the 70 keV-VMI AE, and 0.87 (95% CI: 0.78–0.94) for minimum mean BWIC (pairwise comparison *p* = 0.46 and 0.76). Among patients with NOMI, Youden’s J for diagnosing ITN of the minimum mean BWIC outperformed 50 keV-VMI AE (0.74 [95% CI: 0.54–0.91] *versus* 0.25 [95% CI: 0.00–0.60], *p* = 0.020) and 70 keV-VMI AE (-0.09 [95% CI: -0.22 to 0.00], *p* < 0.001).

Using a two-step approach, first diagnosing NOMI based on the absence of bowel-wall enhancement at 50 keV-VMI and then applying the 0.52 mgI/mL BWIC threshold to abnormal bowel loops to identify ITN among NOMI patients, resulted in the correct classification of 173 out of 177 patients (146 out of 146 without NOMI, 29 out of 31 with NOMI, and 20 out of 23 with NOMI and ITN).

### Comparative performance of BWIC and normalized BWIC for diagnosing NOMI and ITN

For diagnosing NOMI in the overall cohort, absolute BWIC achieved the highest accuracy, with an AUC of 0.98 (95% CI: 0.95–1.00), sensitivity of 97% (95% CI: 83–100), and specificity of 90% (95% CI: 84–95) at the optimal cutoff (Supplementary Table S2). Normalization of BWIC to liver, pancreas, spleen, or a composite “upper abdominal organ” yielded similar or lower AUCs (95% CI: 0.90–0.95) and systematically reduced specificity (77–86%), without statistically significant improvement over absolute BWIC (all non-significant *p*-values, ≥ 0.05). In patients with NOMI, absolute BWIC remained the best-performing metric for detecting ITN in the reader 1 analysis (AUC 0.93 [95% CI: 0.81–1.00]; sensitivity 74% [95% CI: 52–90]; specificity 100% [95% CI: 63–100] at a 0.6 mgI/mL cutoff). Normalized BWIC metrics showed lower AUCs (0.79–0.87) and did not outperform absolute BWIC for ITN detection (all non-significant *p*-values, ≥ 0.05).

## Discussion

This prospective single-center study showed that imaging remained central for diagnosing NOMI, by confirming AE as the most reliable sign. Low-energy 50-keV VMI did not significantly improve AE accuracy despite the highest Youden index. No binary CT feature differentiated ITN from non-ITN, whereas BWIC—evaluated on a dual-layer DECT platform—did (pooled AUC 0.86; Youden J index 0.74 at 0.52 mgI/mL).

A clinically feasible method for BWIC measurement was evaluated across five independent readers in the 31 patients with NOMI. Considering individual measurements as estimates of the same underlying value, pooled analysis identified an optimal threshold of 0.52 mgI/mL for distinguishing ITN from non-ITN. This threshold is remarkably close to the reported iodine detection limit of DECT systems (0.519 mgI/mL) [17], which is physiologically consistent with transmural necrosis, where bowel-wall contrast uptake is essentially absent.

These findings support a pragmatic two-step strategy: first, the careful identification of abnormal bowel-wall enhancement on 50-keV monoenergetic reconstructions for NOMI diagnosis; second, the quantitative assessment of the affected segment by measuring the BWIC using a standardized method, namely the average of five ROIs placed within the abnormal bowel wall [11, 18]. The interobserver reproducibility of the quantitative approach was moderate (absolute-agreement ICC 0.52, 95% CI 0.32–0.70; consistency ICC 0.62, 95% CI 0.47–0.72), but agreement improved when several ROIs were averaged, supporting this multi-ROI approach over single-point sampling. Although the optimal Youden cutoff varied between readers for diagnosing ITN (ranging from 0.36 to 0.65 mgI/mL), diagnostic performance remained high across observers (AUC 0.74–0.93). The higher consistency than absolute-agreement ICC suggests systematic inter-reader offsets rather than purely random variation, emphasizing the need for stricter standardization of ROI placement and platform-specific operational thresholds.

These results require external validation, but the present study already provides a preliminary measurement framework for future studies. In the present cohort, normalizing BWIC to iodine concentration in the liver, spleen, or pancreas did not improve overall diagnostic performance for ITN and generally reduced specificity. This likely reflects the marked variability of solid-organ enhancement in critically ill patients with shock, vasoactive support, and altered contrast kinetics, making organ-based normalization physiologically unreliable in NOMI. Similar limitations have already been reported for aortic normalization [18], while organ-based normalization also increases workflow complexity [19]. In this setting, defining robust, platform-specific absolute BWIC thresholds appears more pragmatic than routine normalization [17, 20].

Beyond imaging, integration with noninvasive biomarkers of intestinal ischemia, such as the intestinal fatty acid–binding protein, which have shown promise for identifying ITN, may further refine stratification of bowel ischemia in NOMI [4, 5, 21]. Overall, the present data support routine DECT use where available, as BWIC uniquely distinguishes ITN from non-ITN. In practice, patients with very low BWIC values (below 0.5 mgI/mL in this cohort) might be considered strong candidates for immediate surgical management, whereas those with higher BWIC values may be candidates for close monitoring and repeat imaging, pending confirmation of these thresholds in larger multicenter studies.

This study has several limitations. First, BWIC measurements were analyzed retrospectively in patients with confirmed NOMI, but data were collected within a prospective cohort with prospectively defined inclusion criteria. Second, external validity is currently restricted to centers equipped with dual-layer DECT, although the technique itself should be transferable to other platforms, with the caveat that optimal thresholds for diagnosing ITN will likely vary across vendors. Third, there is a supposed center effect, as our institution has long-standing expertise in NOMI imaging and management. Surgeons and intensivists in our hospital rely heavily on CT findings for both diagnosis and surgical decision-making, which may partly explain the high diagnostic performance observed for NOMI. We also excluded patients with equivocal CT features in whom the diagnosis of NOMI could not be firmly established, thereby artificially inflating diagnostic performance estimates. Finally, interobserver absolute agreement for BWIC was lower than in a previous study in small-bowel obstruction [11], whereas the reader’s experience of abdominal imaging was low but also likely reflecting the greater complexity of bowel assessment in NOMI. NOMI can involve long, multifocal segments throughout the gut, and because BWIC is segment-dependent [18], readers may not sample exactly the same bowel segment or location, thereby increasing variability. ICU CT scans are also more frequently affected by motion and support-related artifacts. Nevertheless, the higher consistency than absolute agreement suggests systematic inter-reader offsets, underscoring the need to further standardize BWIC measurement and define operational thresholds.

In conclusion, while the BWIC did not outperform VMI for NOMI diagnosis, which already demonstrated excellent accuracy, it uniquely enabled differentiation between ITN and non-ITN. A pragmatic two-step approach combining initial screening with 50-keV-VMI and subsequent BWIC measurement in abnormal segments provides an objective and moderately reproducible framework to stratify NOMI and to guide surgical *versus* conservative management in critically ill patients.

## Supplementary information

**Additional File 1 :** **Table S1** Sensitivity analysis. Diagnostic performance of 5-reader BWICmean for ITN diagnosis in a restricted cohort, with patients without definitive confirmation excluded (*n* = 23 patients: 16 with ITN and 8 without ITN). **Table S2** Diagnostic performance of bowel-wall iodine concentration and normalized iodine metrics for NOMI and irreversible transmural necrosis for Reader 1.

## Data Availability

The datasets generated and/or analyzed during the current study are available from the corresponding author on reasonable request, subject to institutional and regulatory approval.

## Abbreviations

AE: Absent enhancement
BWIC: Bowel wall iodine concentration
CI: Confidence interval
CT: Computed tomography
DECT: Dual-energy computed tomography
ICC: Intraclass correlation coefficient
ICU: Intensive care unit
IQR: Interquartile range
ITN: Irreversible transmural necrosis
NOMI: Non-occlusive mesenteric ischemia
ROI: Region of interest
VMI: Virtual monoenergetic imaging
