# Supplementary material for: DECT-based stratification of nonocclusive mesenteric ischemia using bowel-wall iodine concentration: a prospective single-center cohort
Source: Eur Radiol Exp. 2026 Jun 25;10:100. doi: 10.1186/s41747-026-00760-9 (PMC13305217; doi:10.1186/s41747-026-00760-9)
Supplement: Supplementary file 1 — Additional File 1 : Table S1 Sensitivity analysis. Diagnostic performance of 5-reader BWICmean for ITN diagnosis in a restricted cohort, with patients without definitive confirmation excluded (n = 23 patients: 16 with ITN and 8 without ITN). Table S2 Diagnostic performance of bowel-wall iodine concentration and normalized iodine metrics for NOMI and irreversible transmural necrosis for Reader 1. [file 41747_2026_760_MOESM1_ESM.pdf]

DECT-based stratification of nonocclusive mesenteric ischemia using bowel-wall iodine concentration: a prospective single-center cohort

ELECTRONIC SUPPLEMENTARY MATERIAL

Supplementary Table S1 Sensitivity analysis. Diagnostic performance of 5-reader $BWIC_{mean}$ for ITN diagnosis in a restricted cohort, with patients without definitive confirmation excluded (*n* = 23 patients: 16 with ITN and 8 without ITN)

		AUC	Optimal cutoff*	TP / FP / TN / FN	Sensitivity % (95% CI)	Specificity % (95% CI)	PPV % (95%CI)	NPV % (95%CI)	Accuracy % (95%CI)	Youden J
Reader 1 (4 years of experience)	*$BWIC_{mean}$*	0.89 (0.76–1.00)	≤ 0.60	10 / 0 / 8 / 6	62 (39–82)	100 (68–100)	100 (72–100)	57 (33–79)	75 (55–88)	0.62
	Max Se		≤ 0.89	16 / 5 / 3 / 0	100 (81–100)	38 (14–69)	76 (55–89)	100 (44–100)	79 (60–91)	0.38
	Max Sp		≤ 0.60	10 / 0 / 8 / 6	62 (39–82)	100 (68–100)	100 (72–100)	57 (33–79)	75 (55–88)	0.62
	70 keV-VMI AE	—	—	5 / 2 / 6 / 11	31 (14–56)	75 (41–93)	71 (36–92)	35 (17–59)	46 (28–65)	0.06
	50 keV-VMI AE		—	16 / 6 / 2 / 0	100 (81–100)	25 (7–59)	73 (52–87)	100 (34–100)	75 (55–88)	0.25
Reader 2 (5 years of experience)	*$BWIC_{mean}$*	0.91 (0.79–1.00)	≤ 0.53	16 / 2 / 6 / 0	100 (81–100)	75 (41–93)	89 (67–97)	100 (61–100)	92 (74–98)	0.75
	Max Se		≤ 0.53	16 / 2 / 6 / 0	100 (81–100)	75 (41–93)	89 (67–97)	100 (61–100)	92 (74–98)	0.75
	Max Sp		≤ 0.37	7 / 0 / 8 / 9	44 (23–67)	100 (68–100)	100 (65–100)	47 (26–69)	62 (43–79)	0.44
	70 keV-VMI AE	—	—	12 / 2 / 6 / 4	75 (51–90)	75 (41–93)	86 (60–96)	60 (31–83)	75 (55–88)	0.50
	50 keV-VMI AE		—	16 / 8 / 0 / 0	100 (81–100)	0 (0–32)	67 (47–82)	—	67 (47–82)	0.00
Reader 3 (13 years of experience)	*$BWIC_{mean}$*	0.84 (0.67–1.00)	≤ 0.66	14 / 2 / 6 / 2	88 (64–97)	75 (41–93)	88 (64–97)	75 (41–93)	83 (64–93)	0.62
	Max Se		≤ 0.72	16 / 4 / 4 / 0	100 (81–100)	50 (22–78)	80 (58–92)	100 (51–100)	83 (64–93)	0.50
	Max Sp		≤ 0.46	6 / 0 / 8 / 10	38 (18–61)	100 (68–100)	100 (61–100)	44 (25–66)	58 (39–76)	0.38
	70 keV-VMI AE	—	—	7 / 2 / 6 / 9	44 (23–67)	75 (41–93)	78 (45–94)	40 (20–64)	54 (35–72)	0.19
	50 keV-VMI AE		—	15 / 7 / 1 / 1	94 (72–99)	12 (2–47)	68 (47–84)	50 (9–91)	67 (47–82)	0.06
	$BWIC_{mean}$	0.74 (0.54–0.94)	≤ 0.36	10 / 1 / 7 / 6	62 (39–82)	88 (53–98)	91 (62–98)	54 (29–77)	71 (51–85)	0.50
	Max Se		≤ 0.97	16 / 7 / 1 / 0	100 (81–100)	12 (2–47)	70 (49–84)	100 (21–100)	71 (51–85)	0.12

	Max Sp		≤ 0.26	5 / 0 / 8 / 11	31 (14–56)	100 (68–100)	100 (57–100)	42 (23–64)	54 (35–72)	0.31
Reader 4 (5 years of experience)	*70 keV-VMI AE*	—	—	8 / 3 / 5 / 8	50 (28–72)	62 (31–86)	73 (43–90)	38 (18–64)	54 (35–72)	0.12
	50 keV-VMI AE		—	15 / 6 / 2 / 1	94 (72–99)	25 (7–59)	71 (50–86)	67 (21–94)	71 (51–85)	0.19
Reader 5 (3 years of experience)	*$BWIC_{mean}$*	0.68 (0.45–0.90)	≤ 0.40	8 / 1 / 7 / 8	50 (28–72)	88 (53–98)	89 (56–98)	47 (25–70)	62 (43–79)	0.38
	Max Se		≤ 0.65	16 / 5 / 3 / 0	100 (81–100)	38 (14–69)	76 (55–89)	100 (44–100)	79 (60–91)	0.38
	Max Sp		≤ 0.16	1 / 0 / 8 / 15	6 (1–28)	100 (68–100)	100 (21–100)	35 (19–55)	38 (21–57)	0.06
	70 keV-VMI AE	—	—	8 / 3 / 5 / 8	50 (28–72)	62 (31–86)	73 (43–90)	38 (18–64)	54 (35–72)	0.12
	50 keV-VMI AE		—	16 / 8 / 0 / 0	100 (81–100)	0 (0–32)	67 (47–82)	—	67 (47–82)	0.00
Pooled (5-reader mean)	*Youden*	0.83 (0.66–0.99)	≤ 0.52	13 / 1 / 7 / 3	81 (57–93)	88 (53–98)	93 (69–99)	70 (40–89)	83 (64–93)	0.69
	Max Se		≤ 0.68	16 / 5 / 3 / 0	100 (81–100)	38 (14–69)	76 (55–89)	100 (44–100)	79 (60–91)	0.38
	Max Sp		≤ 0.32	3 / 0 / 8 / 13	19 (7–43)	100 (68–100)	100 (44–100)	38 (21–59)	46 (28–65)	0.19

Data are percentages, with 95% confidence intervals in parentheses. For $BWIC_{mean}$, the optimal cutoff = threshold defined by the Youden index J. Max Se = threshold maximizing sensitivity. Max Sp = threshold maximizing specificity. The pooled analysis = $BWIC_{mean}$ across the 5 readers. *AE* absent enhancement, *AUC* Area under the curve, *BWIC* Bowel-wall iodine concentration, *CI* Confidence interval, *FN* False negative, *FP* False positive, *ITN* Irreversible transmural necrosis, *Max Se* Maximum sensitivity, *Max Sp* Maximum specificity, *NOMI* Non-occlusive mesenteric ischemia, *NPV* Negative predictive value, *PPV* Positive predictive value, *TN* True negative, *TP* True positive, VMI Virtual monoenergetic imaging.

Supplemental Table S2 Diagnostic performance of bowel wall iodine concentration and normalized iodine metrics for NOMI and irreversible transmural necrosis for Reader 1

Diagnosis of NOMI	AUC (CI 95%)	TP / FP / TN / FN	Cutoff	Sensitivity (95% CI)	Specificity (95% CI)	*p*-value *versus* BWIC
$BWIC_{mean}$	0.98 (0.95–1.00)	30 / 14 / 132 / 1	1	97 (83–100)	90 (84–95)	—
$BWIC_{mean}$ normalized on liver	0.94 (0.89–0.97)	30 / 33 / 113 / 1	0.418	97 (83–100)	77 (70–84)	0.486
$BWIC_{mean}$ normalized on pancreas	0.94 (0.89–0.98)	30 / 23 / 123 / 1	0.383	97 (83–100)	84 (77–90)	0.577
$BWIC_{mean}$ normalized on spleen	0.90 (0.81–0.97)	25 / 28 / 116 / 3	0.358	89 (72–98)	81 (73–87)	0.299
$BWIC_{mean}$ normalized on upper abdominal organs	0.95 (0.90–0.99)	30 / 20 / 126 / 1	0.36	97 (83–100)	86 (80–91)	0.713

Diagnosis of ITN in patients with NOMI	AUC (CI95%)	TP / FP / TN / FN	Cutoff	Sensitivity (95% CI)	Specificity (95% CI)	*p*-value *versus* BWIC
$BWIC_{mean}$	0.92 (0.81–1.00)	17 / 0 / 8 / 6	0.6	74 (52–90)	100 (63–100)	—
$BWIC_{mean}$ normalized on liver	0.79 (0.59–0.95)	15 / 1 / 7 / 8	0.214	65 (43–84)	88 (47–100)	0.517
$BWIC_{mean}$ normalized on pancreas	0.87 (0.71–0.98)	15 / 0 / 8 / 8	0.194	65 (43–84)	100 (63–100)	0.793
$BWIC_{mean}$ normalized on spleen	0.81 (0.63–0.95)	16 / 0 / 6 / 6	0.218	73 (50–89)	100 (54–100)	0.620
$BWIC_{mean}$ normalized on upper abdominal organs	0.86 (0.69–0.98)	18 / 1 / 7 / 5	0.265	78 (56–93)	88 (47–100)	0.778

AUC Area under the curve, *BWIC* Bowel wall iodine concentration, *FN* False negative, *FP* False positive, *NOMI* Non-occlusive mesenteric ischemia, *TP* True positive, *TN* True negative.
